# Efficacy of a new-generation platelet-rich fibrin in the treatment of periodontal intrabony defects: a randomized clinical trial

**DOI:** 10.1186/s12903-021-01925-1

**Published:** 2021-11-15

**Authors:** Boróka Klára Csifó-Nagy, Eleonóra Sólyom, Vera Lili Bognár, Annamária Nevelits, Ferenc Dőri

**Affiliations:** Department of Periodontology, Faculty of Dentistry, Semmelweis University, Szentkirályi u. 47., Budapest, 1088 Hungary

**Keywords:** Intrabony defects, Periodontal healing, Advanced platelet-rich fibrin, Enamel matrix derivative

## Abstract

**Background:**

The aim of the study was to clinically evaluate the healing of intrabony defects after treatment with a new generation of platelet-rich fibrin (A-PRF+) respect to enamel matrix derivative (EMD).

**Methods:**

Thirty (30) intrabony defects of 18 patients (9 males, 9 females) were randomly treated with A-PRF+ (test, n = 15) or EMD (control, n = 15). The following clinical parameters were recorded at baseline and 6 months after surgery: pocket depth (PD), gingival recession (GR) and clinical attachment level (CAL). After debridement the intrabony defects were filled with A-PRF+ in the test group, respectively with EMD in the control group, and fixed with sutures to ensure wound closure and stability.

**Results:**

Both treatment methods resulted in statistically significant PD reductions, respectively CAL gains six months post-operatively. No statistically significant differences were found between the two groups as the mean CAL gain was 2.33 ± 1.58 mm in the A-PRF+ group, respectively 2.60 ± 1.18 mm in the EMD group (*p* < 0.001).

**Conclusion:**

Within the limits of this study the new-generation platelet-rich fibrin seems to be as clinically effective as EMD during surgical treatment of intrabony defects. Treatment with A-PRF+ or EMD resulted in reliable clinical outcomes. The use of A-PRF+ as a human autologous product can give a positive impact on periodontal healing.

*Clinical Relevance* A-PRF+ may be suitable for the treatment of intrabony periodontal defects.

*Trial registration number (TRN)* NCT04404374 (ClinicalTrials.gov ID).

## Background

The main goal of the comprehensive periodontal treatment is to eliminate inflammation and to prevent further destruction of the periodontium, as well as to achieve sustainable condition in the long-term [1]. Over the past decades, significant efforts have been made to develop both materials and surgical techniques that can predictably contribute to periodontal regeneration [2]. Nowadays, regenerative periodontal therapy can restore only a part of the original tissue to some extent, while complete periodontal restoration remains idealistic [3]. Enamel matrix derivative (EMD) was introduced to enhance periodontal regeneration by mimicking the formation of periodontal attachment tissues more than 20 years ago. They play an important role as chemical barriers and biological mediators in periodontal regeneration and healing [4]. Advances in cell and molecular biology have contributed to an increased understanding of wound healing. There is evidence that polypeptide growth and differentiation factors (GDF’s) can support wound healing and regeneration by regulating chemotaxis, differentiation, proliferation of cells and matrix synthesis. However, only a few factors have reached clinical evaluation, earlier research objectives expected only to achieve the optimal dosage and the combination of growth and differentiation factors [5]. Data from long-term follow-up clinical studies demonstrated that treatment of deep periodontal intrabony defects with EMD resulted in a significant increase of clinical attachment gain and bone fill compared to’open flap debridement’ [6–9]. Introduction of autologous platelet concentrates (Platelet-Rich Plasma—PRP, Platelet-Rich Gel—PRG, Platelet-Rich Fibrin—PRF) presents a new period in periodontal application of chemical-biological factors [10–14].

A strategy to promote wound healing is to amplify and accelerate the effect of released growth factors (GF’s), which can accelerate the healing of bone defects and promote periodontal regeneration. The simplest way to achieve these goals is to activate the local release of platelet-derived growth factors, which are common triggers in almost all wound healing processes [15]. A method for the intraoral application of concentrated autologous products (CAP) has been developed for more than two decades. The use of PRP is based on the effect of growth factors released from concentrated platelets on healing and tissue regeneration [16, 17].

Due to advances in platelet-rich concentrate formulations over the past decade, PRF was introduced and has been used as a supra-physiological concentration of autologous growth factors, without the addition of anticoagulants [14, 18]. The PRF is a product that enhances wound healing, unlike PRP. The beneficial effects of PDGF’s, TGF-beta (platelet-derived growth factor, transforming growth factor beta) and other GF’s released from platelets can be recorded not only in the early stages of wound healing, but also last longer and appear at a slower rate [10, 19].

Choukroun et al. introduced Advanced Platelet Rich Fibrin (A-PRF), a new generation of platelet-rich fibrin formulation obtained by a lower speed centrifugation in 2014 [20].

Standard leukocyte-rich PRF (L-PRF) and A-PRF obtained through the experimental’low-force modified procedure’ are ideal sources of leukocytes that act directly on the release of chemokines and growth factors. Although A-PRF ‘traps’ as many leukocytes as L-PRF does and releases the same amount of inflammatory cytokines, it contains higher amounts of PDGF and VEGF [21]. Another study found that additionally A-PRF contained significantly more TGF-β1, EGF, and IGF, and showed significantly higher human fibroblast migration and proliferation than L-PRF. The same study suggests that reducing the centrifugation rate favours the growth factors released from the PRF clot [22]. As it was described by Fujioka-Kobayashi et al., A-PRF+ product that is the new version of A-PRF (2017) needs not only lower centrifugation speed, but also less time (1300 rpm for 8 min) and demonstrates an increased growth factor release of TGF-beta1, PDGF-AA, PDGF-AB, PDGF-BB, VEGF, IGF, as well as EGF. When compared to L-PRF and A-PRF, A-PRF+ demonstrated a significant increase in growth factor release within either 1, 3, or 10 days [22]. In conclusion, results show that the total growth factor release could be enhanced by reducing both centrifugation speed and time in A-PRF+. In the course of the examination of the fibrin network in terms of structural integrity, A-PRF+ showed similar porosity to A-PRF’s, furthermore the cellular distribution pattern showed evenly dispersed platelets over the entire clot. These observations emphasize the improved regenerative capacity of advanced PRF matrices.

The research and application of EMD as practically non-human biological mediators (Emdogain^®^, Straumann^®^, Basel, Switzerland) in periodontal regenerative surgery, although dating back more than two decades still raise several unanswered or ambiguously answered questions. Research on the use of autologous growth and differentiation factors as well as recombinant growth factors (rhGF’s) as biological mediators in periodontal regenerative procedures has a relatively shorter history and offers many additional opportunities for researchers. Examining the new generation of PRF is an even more topical task.

## Methods

Our aim was to investigate the further roles of human autologous platelet concentrates in periodontal healing and regeneration. We tested the role and clinical applicability of a new generation of platelet-rich fibrin,’Advanced Platelet-Rich Fibrin (A-PRF+)’ in periodontal wound healing. Clinical data obtained during the study were compared with the results of a well-known and successfully applied regenerative method, since two decades of experience with enamel matrix derivative are available [23]. The null hypothesis of the study was whether an autologous material (A-PRF+) can be a reliable alternative in surgery of intrabony defects.

### Study design

This study was planned as a randomized, controlled, prospective clinical trial, performed in accordance with the Helsinki Declaration of 1975, as updated in 2013 [24], and the protocol was approved by the Ethics Committee of the Semmelweis University Budapest (SE TUKEB: 254/2017). The study protocol was retrospectively registered at ClinicalTrials.gov with ID number NCT04404374.

The study was conducted at the Department of Periodontology, Semmelweis University, Budapest, Hungary. It was initiated in June 2018 and completed in November 2019 by the same experienced periodontist (BKCSN). Risks, benefits, and procedure were explained to each participant in their native language and a written informed consent was obtained.

### Study population

According to the new classification proposed by the World Workshop on the Classification of Periodontal and Peri-Implant Diseases and Conditions (2017), the patients were classified into stage III. periodontitis [25]. At baseline the patients received cause-related periodontal therapy, consisting of oral hygiene instruction, motivation and sub-gingival scaling/root planing under local anaesthesia. The patients were consecutively enrolled when the following inclusion criteria were met: (1) no systemic diseases, like diabetes mellitus, osteoporosis, immunosuppressed conditions that may affect the outcome of the therapy; (2) no smoking [26, 27]; (3) good level of oral hygiene with Full-Mouth Bleeding Score < 20% [28] and Full- Mouth Plaque Index Score < 20% [29]; (4) presence of a minimum one or more 2-, 3-, or combined 2–3-wall intrabony defect with a defect angle of 20–40 (± 5) degrees, as the radiographic defect angle influences the outcome of regenerative surgical therapy in intrabony defects [30] and with a minimum probing depth (PD) of 6 mm and intrabony component of a minimum 4 mm.

### Clinical parameters

The following clinical parameters were assessed at baseline (1 week before surgery) and at six months after the surgery, using the same type of periodontal probe (UNC-15, Hu-Friedy, Chicago, IL, USA): Full-Mouth Plaque Score (FMPS) [29] and Full-Mouth Bleeding Score (FMBS) [28], pocket depth (PD), gingival recession (GR), clinical attachment level (CAL) and transgingival bone sounding (BS). The primary outcome was CAL gain. Radiographs were performed with’long cone’ technique before surgery, and 6 month post-surgically. The clinical measurements were made at six sites per tooth, mesio-buccal, mid-buccal, disto-buccal, mesio-lingual, mid-lingual, and disto-lingual, by the same experienced, calibrated investigator (ES), and the highest PD value was taken into consideration.

### Blinding and calibration

The examiner was not aware of the type of treatment rendered in any of the cases. The measurements were rounded up to the nearest millimeter.

To calibrate the examiner five patients were used, each showing 10 teeth (single and multi- rooted) with PDs > 6 mm on at least one aspect of each tooth. The examiner evaluated the patients on two separate occasions, 48 h apart. Calibration was accepted if > 90% of the recordings could be reproduced within 1.0-mm difference.

### Randomization

The defects were randomly allocated by computerised random number generator (https://www.sealedenvelope.com) and treated with either A-PRF+ (test group) or EMD (control group).

### Preparation of A-PRF+

Immediately before surgery A-PRF+ was prepared for the test group using a commercially available PRF Kit [Process for PRF^®^ (A-PRF), J. Choukroun, Nice, France] and ‘Process for PRF Duo’ (Choukroun) centrifuge. Cubital venous blood was drawn from the patient without the addition of anticoagulant into two 10 ml vacuum tubes (A-PRF+ tube, Choukroun 2017) and immediately centrifuged at 1300 rpm for 8 min, then allowed to rest for 5 min [22]. During centrifugation, 3 layers are formed in the tube at the start of the coagulation cascade. The top layer is Platelet-Poor Plasma (PPP), the middle layer is Platelet-Rich Fibrin’clot’ (PRF) is to be used, and the bottom one is the Red Blood Cells (RBC’s) layer. The PRF’clot’ still in gel condition is removed from the tube, cleaned of red blood cells and used as a gel.

### Surgical procedure

**Fig. 1:**
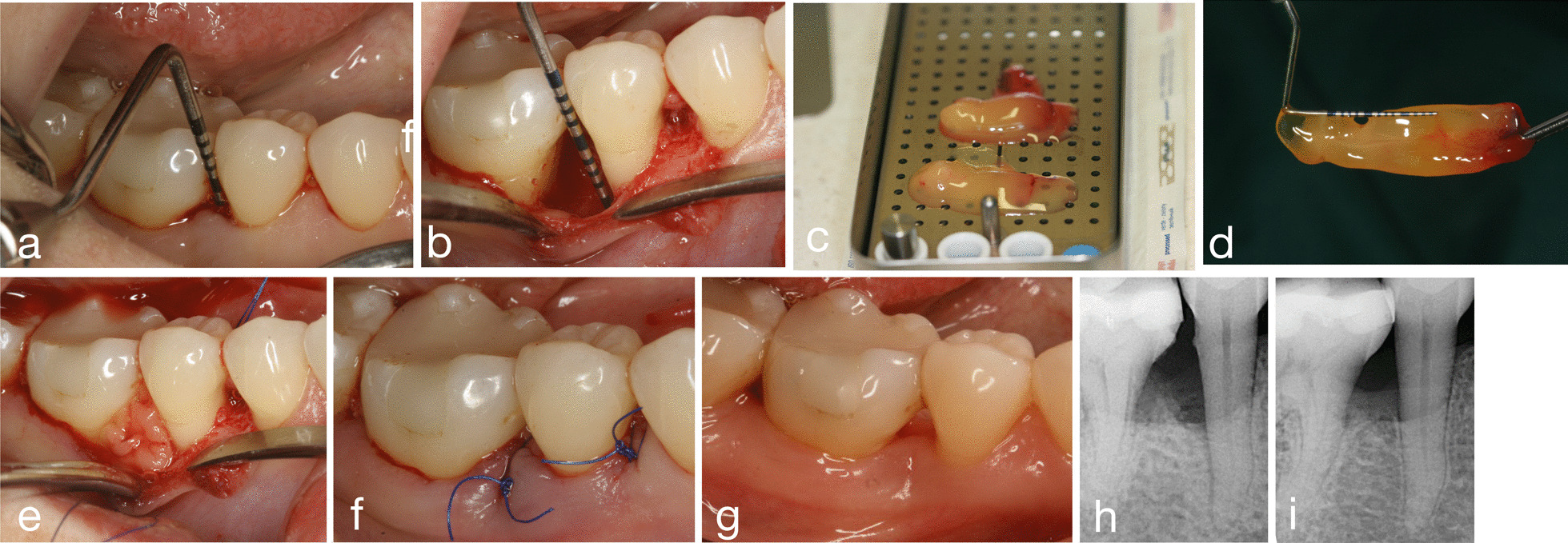
Treatment of an intrabony defect at a lower jaw premolar with A-PRF+. **a** Preoperative measurements, **b** defect after debridement—intraoperative measurements, **c** product after centrifugation in the PRF Box (Process for PRF^®^), **d** prepared product (RBC + A-PRF+), **e** intrabony defect filled with A-PRF+, **f** wound closure, **g** 6 months after surgery, **h**, **i** radiographic evaluation before and 6 months after surgery

**Fig. 2:**
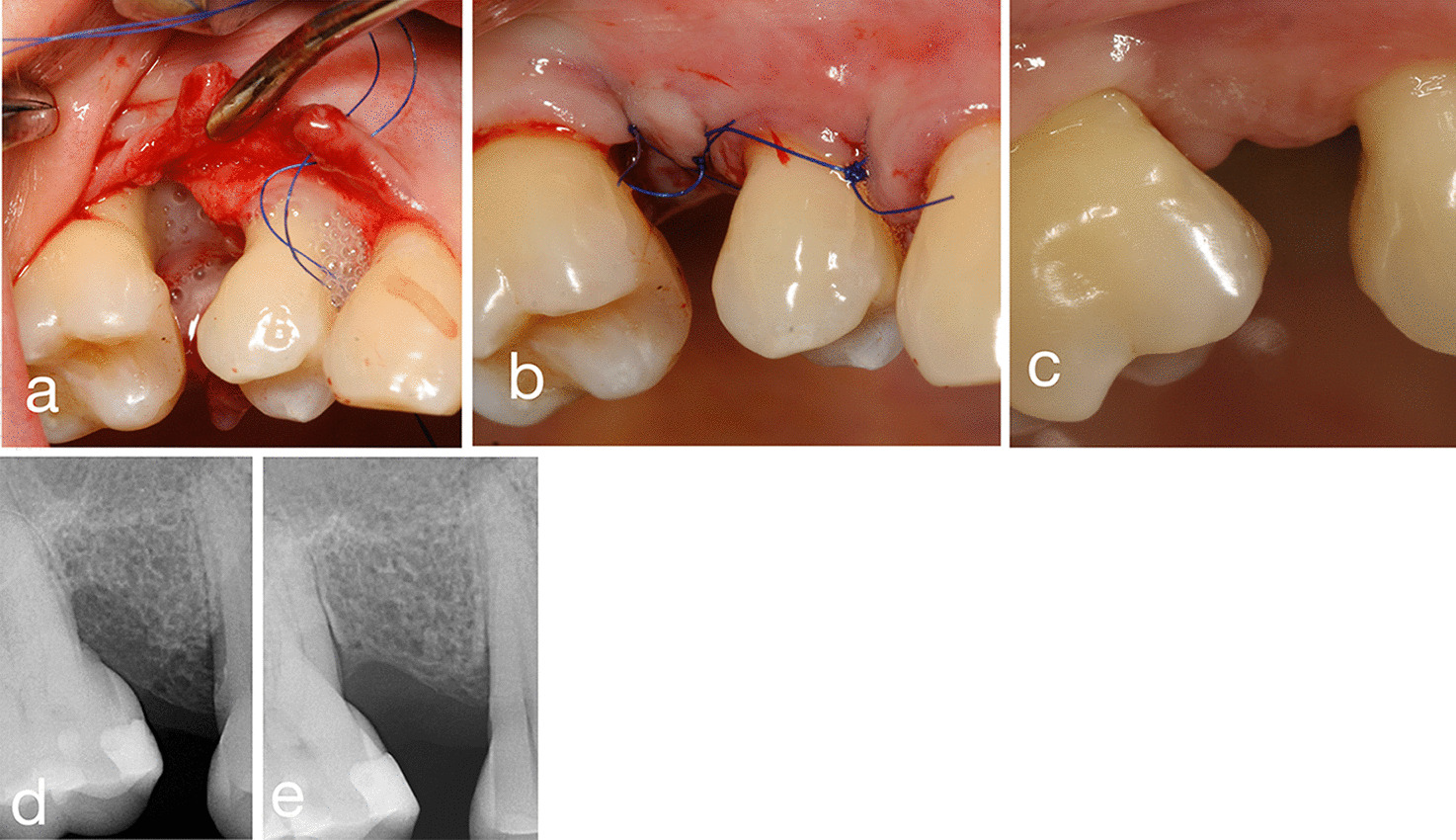
Treatment of an intrabony defect at an upper jaw molar with EMD. **a** Defect after debridement—intrabony defect filled with EMD, **b** Wound closure, **c** 6 months after surgery, **d**, **e** radiographic evaluation before and 6 months after surgery

Surgery was performed by a single experienced operator, specialist in periodontics (BKCSN). After administration of local anesthesia intracrevicular incisions were performed extending to the adjacent teeth, with additional precaution to preserve the maximum of interdental gingival tissue. Full-thickness buccal and oral extended flaps were raised, and all granulation tissue was removed from the defect without bone recontouring. The roots were thoroughly scaled and planed by means of hand and ultrasonic instruments. After defect debridement, A-PRF+ was applied in the test group (Fig. 1). In the control group after debridement the root surface adjacent to the defect was conditioned for 2 min with 24% ETDA gel (pH 6.7) (PrefGel, Straumann^®^, Basel, Switzerland) [31]. The defect and the adjacent mucoperiosteal flap were then thoroughly rinsed with sterile saline to remove all EDTA residue, and then Straumann^®^ Emdogain^®^ was applied (Fig. 2). Finally, the flap was repositioned coronally and closed thoroughly with 5.0. non-absorbable modified vertical or horizontal mattress sutures (Dafilon^®^ 5.0. monofilament and uncoated polyamide, B. Braun Surgical S.A. Barcelona, Spain).

### Postoperative care

All the patients had received antibiotics for a week, two times daily (Augmentin Duo, 875 mg amoxycillin/125 mg clavulanic acid, GlaxoSmithKline, Brentford, United Kingdom) [32]. The postoperative care consisted of 0.2% chlorhexidine (Curasept ADS 220, Curaden AG, Kriens, Switzerland) rinses two times daily for 3 weeks. Patients were advised not to use mechanical means of plaque control in the surgical area for more weeks. Sutures were removed 14 days after surgery. Instructions for maintenance of proper oral hygiene were reinforced. Study participants were scheduled for follow-up visits weekly for one month post-surgery and subsequently at three and respectively six months interval.

### Statistical analyses

For the clinical parameters, data were evaluated using descriptive analysis with results illustrated as mean ± SD, range at baseline and 6 months interval. The statistical package Stata (StataCorp. 2017. Stata Statistical Software: Release 15. College Station, TX: StataCorp LLC) was used for data handling and analysis.

Defects identified (rather than patients) were treated as the unit of observation. Generally, two samples of 15 observations each adequately powered (80%) to detect a between-groups difference of 1.06 standard deviations (SD) in a continuous variable, assuming equal SDs across groups. Post-hoc power calculations were carried out for between-groups comparisons at 6 months and within-group comparisons (6 months vs baseline) for each outcome. Within-group changes were evaluated using paired t-tests (or Wilcoxon’s matched-pairs signed-ranks test if parametric assumptions were not satisfied), and between-groups comparisons were made using two-sample t-tests (or Wilcoxon’s rank-sum tests if parametric assumptions were not satisfied). *p* values < 0.05 were considered to indicate statistical significance. Regarding frequency distribution changes from baseline to 6 months were categorized in each outcome as decrease, no change, and increase. To compare the two groups in terms of these, Fisher’s exact test was used (Table 1).

**Table 1:** Frequency distribution of the results (intergroup comparison, *p* > 0.999)

	A-PRF+	EMD	Total
*PD*			
Decrease	15	15	30
Total	15	15	30
*GR*			
Decrease	0	1	1
No change	5	4	9
Increase	10	10	20
Total	15	15	30
Fisher's exact			1.000
*CAL*			
Decrease	13	15	28
No change	2	0	2
	15	15	30
Fisher's exact			0.483
*BS*			
Decrease	14	14	28
No change	1	1	2
	15	15	30
Fisher's exact			1.000

*PD* Probing depth, *GR* gingival recession, *CAL* clinical attachment level, *BS* bone sounding

## Results

### Participants and recruitment

**Fig. 3:**
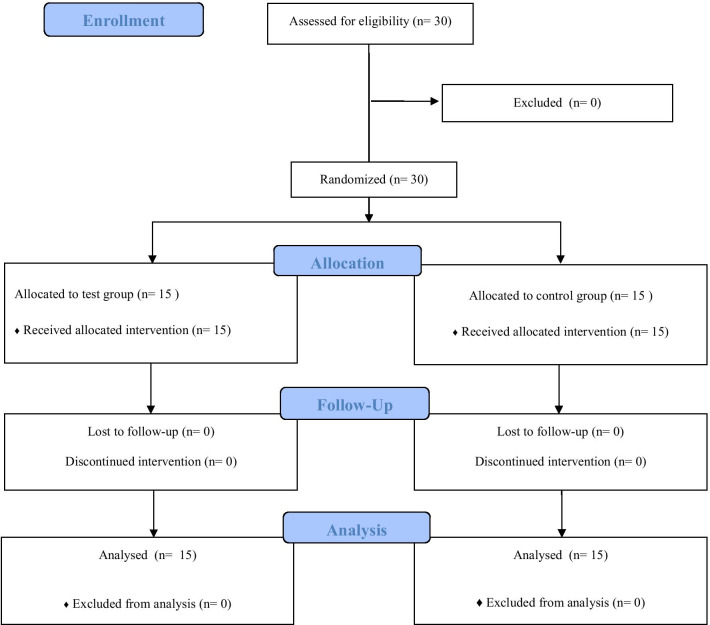
Flow diagram of patient enrollment and study process

The study flowchart is illustrated in Fig. 3. This study has enrolled thirty (30) intrabony defects of 18 non-smoking patients (nine males and nine females) age 55.5 ± 14.5 years, suffering from chronic periodontitis. Among patients the defect distribution was proportional. Baseline and postoperative FMBS and FMPS values were comparable, FMBS values decreased after surgery (Table 2). Post-hoc power calculations for between-groups comparison of PD and BS values were not estimable because the two group means at 6 months were identical. For GR and CAL values, there was an estimated 11% power. For within-group comparisons, power estimates of at least 99.8% were obtained for all outcomes.

**Table 2:** Baseline and postoperative FMBS and FMPS values

	A-PRF+ (%)	EMD (%)
*FMBS*		
Baseline	23	22
6 months postop	10	12
*FMPS*		
Baseline	17	18
6 months postop	16	19

*FMBS* Full-mouth bleeding score, *FMPS* full-mouth plaque score

All of the patients completed the study and post-operative recovery was uneventful without exception. No complications such as allergic reactions or infections were identified throughout the entire study period. The trial was terminated after the completion of the six-month follow-up and analysis of the data of 30 intrabony defects, which displayed a comparable distribution and configuration in the two groups (Table 3).

**Table 3:** Distribution and configuration of treated defects

	A-PRF+	EMD
*Tooth location*		
Maxilla	6	7
Mandible	9	8
Anterior teeth	4	3
Premolars	6	5
Molars	5	7
*Defect configuration*		
2-wall	4	3
3-wall	4	4
2–3-wall combined	7	8

No statistically significant differences were found between the two groups regarding the mean values of the baseline clinical parameters. After 6 months, the mean PD has decreased significantly in both groups compared to baseline data (*p* < 0.001). The mean PD reduction was 4.67 ± 0.62 mm in the A-PRF+ group and identically 4.67 ± 0.62 mm in the EMD group. No statistically significant difference between the groups was found (Table 4).

**Table 4:** Within group comparisons/intergroup changes (significance: *p* < 0.05)

	Baseline	6 months postop	*p*	Diff	*p*
*PD*					
A-PRF+	8.27 ± 1.58	4.67 ± 0.62	*p* < 0.0001	−3.6 ± 1.68	*p* = 0.0000
EMD	8.13 ± 1.60	4.67 ± 0.62	*p* < 0.0001	−3.46 ± 1.30	*p* = 0.0000
		*p* = 0.999			
Cohen's d		d = −0.092			
*GR*					
A-PRF+	2.67 ± 1.88	3.93 ± 2.73	*p* < 0.0025	1.26 ± 1.33	*p* = 0.0025
EMD	2.47 ± 1.46	3.33 ± 1.58	*p* < 0.0044	0.86 ± 0.99	*p* = 0.0044
		*p* = 0.,469			
Cohen's d		d = 0.352			
*CAL*					
A-PRF+	10.93 ± 2.7	8.6 ± 2.56	*p* < 0.0001	−2.33 ± 1.588	*p* = 0.0001
EMD	10.60 ± 1.76	8.00 ± 1.77	*p* < 0.0001	−2.6 ± 1.18	*p* = 0.0000
		*p* = 0,461			
Cohen's d		d = 0.197			
*BS*					
A-PRF+	9.60 ± 1.68	5.67 ± 0.89	*p* < 0,0001	−3.93 ± 1.98	*p* = 0.0000
EMD	9.47 ± 1.68	5.67 ± 0.81	*p* < 0,0001	−3.8 ± 1.56	*p* = 0.0000
		*p* = 0.999			
Cohen's d		d = −0.077			

*PD* Probing depth, *GR* gingival recession, *CAL* clinical attachment level, *BS* bone sounding

After 6 months, the mean GR increase was 3.93 ± 2.73 mm in the test group and 3.33 ± 1.58 mm in the control group. The increase in GR was statistically significant for both groups (*p* < 0.01), but no difference between the groups was observed.

The mean CAL gain was 2.33 ± 1.58 mm in the A-PRF+ group and 2.60 ± 1.18 mm in the EMD group (*p* < 0.001). In both groups, the CAL has improved significantly compared to baseline, but no statistically significant difference was found between the two groups.

After 6 months, the mean BS has decreased to 5.67 ± 0.89 mm in the test group and to 5.67 ± 0.81 mm in the control group. Compared to baseline data 9.60 ± 1.68 mm in the test group, respectively 9.47 ± 1.68 mm in the control group, the mean BS reduction was significant (*p* < 0.001), but no difference between the groups was observed, the results obtained with either material were similar (Table 4).

## Discussion

One of the main benefits of PRF is the fibrin network which promotes not only blood clot formation but also tissue repair mechanisms [33]. Compared to PRP, the kinetics of growth factor release appear to be slower, thus affecting regeneration over a longer period of time [34]. More and more studies are drawing attention to the beneficial effect of leukocytes on healing, on tissue regeneration, and not least to the importance of the quality of the fibrin network. The leukocytes it contains have both anti-infective and immunoregulatory functions [35–38], but also produce significant amounts of VEGF [39]. These factors, in addition to platelet-derived angiogenesis-stimulating factors, may have a positive impact on proper blood supply of the healing wound. White blood cells are involved in the early stimulation of osteo-progenitor cells and promote the differentiation of monocytes into macrophages [18, 20, 39–41].

Many controlled randomized clinical trials investigated the use of PRF for the repair/regeneration of periodontal intrabony defects [42–46]. All of these studies demonstrated that the additional application of PRF increased PD reductions and CAL gains compared to open flap debridement alone. In a recent publication the supplementation of PRF with EMD did not result in a difference between the study and control (EMD only) groups [47]. The efficacy of PRF and EMD in the treatment of intrabony defects was compared in a clinical and a cone beam computed tomography study. Based on the obtained results, both materials were effective in the treatment of intrabony defects, however EMD was significantly superior in terms of percentage defect resolution [48]. Although these clinical trials have all shown that the use of PRF results in statistically significant CAL gains and PD reduction, it is important to emphasize that histological examination would be necessary to confirm whether the obtained results correspond to a periodontal regeneration or a periodontal repair.

It has been demonstrated that the biological benefits of PRF act locally by rapidly stimulating a large number of cell types by influencing their recruitment, proliferation and/or differentiation [17]. Based on the available literature, it seems that PRF favours the regeneration of soft tissues rather than hard tissues [49]. In the treatment of intrabony defects where space maintenance is not an issue, blood clot formation alone might be sufficient [50], the additional use of PRF acts primarily as a scaffold and may promote tissue regeneration when inserted into the periodontal pocket [19]. Further research is needed to determine which factors in the PRF clots (cells/leukocytes, growth factors, or fibrin matrix) are most required to accelerate the regeneration of periodontal tissues.

Data from in vitro studies indicate that EMD may have an impact on periodontal wound healing by an indirect stimulatory effect on the release of growth factors during periodontal healing and by inhibiting or at least retarding epithelial down-growth [51].

The modification of the preparation protocol by reducing the applied centrifugation force (RCF), resulted in an improved preparation protocol for advanced PRF (A-PRF) using 208 g RCF. Compared to PRF the A-PRF clot showed a more porous structure with larger interfibrous space, where cells (particularly platelets) were observed in even distributions throughout the entire clot, furthermore histological analysis of A-PRF has showed a significantly higher number of neutrophile granulocytes [49].

Fujioka-Kobayashi et al. found and described that the total growth factor release could be enhanced by reducing both centrifugation speed and time. A-PRF+ showed similar porosity to A-PRF, furthermore the cellular distribution pattern that it showed evenly dispersed platelets over the entire clot. These observations emphasize the improved regenerative capacity of advanced PRF matrices [22].

The results of the present study obtained after six months post-surgically clearly indicate the significant improvement of the following parameters: PD, CAL, BS in both groups. No adverse reactions had been observed throughout the first six months, which clearly indicates that the use of autologus test material has been well tolerated.

As a result of surgery and cessation of inflammation, the rate of gingival recession became significantly higher in both groups compared to the baseline. Upon intergroup comparison the GR increase was found to be non-significantly different. The cause of gingival recession observed after periodontal surgery does not necessarily depend on the methods. However, the gingival biotype can significantly affect the extent of the recession. At the same time, a significant improvement in clinical probing pocket depth resulted in a significant enhancement of the clinical attachment level. In addition to the significant improvement of bone sounding values, radiographs taken with the’long-cone’ technique also suggest the presence of bone filling. (Figs. 1, 2.)

It has been two decades since the introduction of EMD, which still remains one of the most successful methods for periodontal regeneration, however anatomical factors like defect configuration seem to play an important role in EMD-induced periodontal healing [23]. The presented results obtained in the control group are in concordance with other controlled clinical trials, which have proven in the long term that treatment of intrabony defects with EMD may result in significantly higher CAL gains and PD reductions [52], in addition no treated teeth were lost during the observation period [7].

There was no significant difference between the test and control groups in the first 6 months after surgery and the results obtained with either materials were similar. The amount of gingival recession seems to be lower in the control group. Thus, it appears that the clinical benefit of both treatments is the improvement of PD values by facilitating plaque control and maintenance. However, when interpreting these findings, we must keep in mind that currently no other data evaluating the treatment of intrabony defects with A-PRF+ versus EMD are available. Therefore, direct comparisons with other studies are not possible at this point in time.

When interpreting the obtained healing results, attention must also be paid to the fact, that the study design was double-arm without a control group for direct comparison. This factor may be a limitation of the study, as isolated intrabony defects may present bone filling without the addition of biomaterials [50].

On the other hand, it also needs to be considered that the lack of difference between the two groups could be attributed additionally to the rather limited number of treated defects (e.g. 15 defects in each group) and therefore, the study may not have the statistical power to rule out the possibility of a difference between the two groups. It is estimated that a sample size of about 30 persons per group would be needed for superiority trials, in the treatment of periodontal intrabony defects [53].

Finally, the present study using a new generation of PRF seems to open new horizons in the investigation of the effects of platelet-concentrates on periodontal healing. However, other randomized, clinical trials with a bigger population and with histological evaluation of periodontal regeneration will be necessary to confirm the results of this study.

## Conclusion

In view of the above findings and within the limits of the current study, the results indicate that the new-generation platelet rich fibrin behaves as effectively as enamel matrix derivative in the surgical treatment of intrabony periodontal defects. Based on the 6-month results, both methods resulted in comparable outcomes and presented no significant differences between the test and control groups. A-PRF+ seems to be suitable for the treatment of intrabony periodontal defects.

## Data Availability

Authors can confirm that all relevant data are included in the article and/or its supplementary information files.

## Abbreviations

A-PRF: Advanced platelet-rich fibrin
EMD: Enamel matrix derivative
PRP: Platelet-rich plasma
PRG: Platelet-rich gel
PRF: Platelet-rich fibrin
GDF’s: Growth and differentiation factors
CAP: Concentrated autologous products
PDGF: Platelet-derived growth factor
TGF-β: Transforming growth factor beta
L-PRF: Leukocyte- and platelet-rich fibrin
EGF: Epidermal growth factor
IGF: Insulin-like growth factor
VEGF: Vascular endothelial growth factor
rhGF: Recombinant human growth factors
PD: Probing depth
GR: Gingival recession
CAL: Clinical attachment level
BS: Bone sounding
FMBS: Full-mouth bleeding score
FMPS: Full-mouth plaque score
EDTA: Ethylenediamine tetraacetic acid
